# Peripheral Neuritis Due to Sulphide of Carbon

**Published:** 1910-04-02

**Authors:** 


### Medicine.

### PERIPHERAL NEURITIS DUE TO SULPHIDE OF CARBON.

Organic compounds of various kinds are now employed in all sorts of trades, such as those concerned with the manufacture of indiarubber and so forth, and an increasing number of cases of peripheral neuritis due to one or other is being recorded; their importance from the point of view of employers' liability is obvious.

The following is a case illustrative of peripheral neuritis due to sulphide of carbon. The patient is a girl of 17, of small stature, who came into the hospital for emaciation, extreme weakness, and anæmia. The illness had begun somewhere about two years before, when she had come to town and taken up her lodging in a badly ventilated room which she shared with her sister. Besides general symptoms of ill-health she experienced a great falling off in her appetite, shortness of breath on exercise, and dysmenorrhœa. She had been employed in a factory where she made indiarubber balls, her duty being to steep the balls in a bath of sulphide of carbon and blow them up with a bellows.

Hardly a month after she had begun this work her sense of tiredness increased; she became thin; the menstruation ceased completely, and was replaced by persistent leucorrhœa. There was neither headache, nor epistaxis, nor loss of consciousness; she often complained, however, of coldness of her extremities, of pains in her back, and of painful spots on her flanks. Presently she noticed that her right hand was getting feeble, and that when she wanted to carry things she could scarcely do so, and this impairment of strength in the right upper limb became so marked that she came to the hospital. When seen both her hands presented an abnormal red colour owing to staining at her work. Her viscera gave no abnormal signs, except that there was a hæmic bruit over the heart. There was no pyrexia. As regards the nervous system, the first point to attract attention was a vasomotor disturbance in her extremities, especially in her right hand, associated with lividity and coldness. There was very marked paresis of the muscles of the right hand and of all the flexors in the right forearm; although the left was probably affected too, it was so much stronger than the right that it seemed to be relatively normal.

There were sensory changes also; great impairment of cutaneous sensibility extended up on to the right forearm for 12 cm. on the radial side, and 15 cm. on the ulnar side. Above this level there was more sensibility, but not normally until above the elbow. The patient was unable to distinguish objects by their shape when she felt them with her right hand, yet muscle sense was retained. There was no impairment of sensation in the left arm, and the lungs were healthy. The patient had a good intelligence, and was in no way hysterical. Under treatment by means of hot baths, electricity, and medicines the paræsthesia gradually diminished, but it was a considerable while before the patient was anything like cured.

